# A Mathematical Model for Storage and Recall of Images using Targeted Synchronization of Coupled Maps

**DOI:** 10.1038/s41598-017-09440-6

**Published:** 2017-08-21

**Authors:** P. Palaniyandi, Govindan Rangarajan

**Affiliations:** 1 Department of Physics, Nehru Memorial College, Puthanampatti, Tamil Nadu India; 2 Department of Mathematics and Centre for Neuroscience, Indian Institute of Science, Bangalore, India

## Abstract

We propose a mathematical model for storage and recall of images using coupled maps. We start by theoretically investigating targeted synchronization in coupled map systems wherein only a desired (partial) subset of the maps is made to synchronize. A simple method is introduced to specify coupling coefficients such that targeted synchronization is ensured. The principle of this method is extended to storage/recall of images using coupled Rulkov maps. The process of adjusting coupling coefficients between Rulkov maps (often used to model neurons) for the purpose of storing a desired image mimics the process of adjusting synaptic strengths between neurons to store memories. Our method uses both synchronisation and synaptic weight modification, as the human brain is thought to do. The stored image can be recalled by providing an initial random pattern to the dynamical system. The storage and recall of the standard image of Lena is explicitly demonstrated.

## Introduction

In this paper, we propose a mechanism for storage and recall of images that mimics the mechanisms used in the human brain. The mechanisms by which the human brain stores and recalls memory^1^ are still an active area of research. Two important mechanisms, which we focus on, include modification of synaptic weights^1^ and synchronization^2–4^. In this work, we propose a model that uses both of these mechanisms whereas prior models primarily used modification of synaptic weights. Existing mathematical models include neural network models^1, 5–7^, Gabor filters in the context of the visual system^8^ etc. In this paper, we investigate a model using coupled maps that provides an explicit method to store and recall visual images. We use Rulkov maps that have been extensively used earlier for modeling neurons^9^. Rulkov map is a 2-d map where one of the variables represents the membrane potential. By varying the parameters of the model, one can get different states of the neuron like spiking and chaotic bursts. Our model is, however, independent of the specific map that is used. We show, given an input image, it can be stored as a pattern of coupling strengths mimicking the patterns of synaptic weights that are hypothesized to be used in memory encoding. The storage and recall mechanism uses a technique that is a generalization of ‘targeted synchronization’, a particular case of synchronization. In targeted synchronization, only a desired (targeted) subset of maps is synchronized and this builds on the earlier work of Chen *et al*.^10^ on generalized Turing patterns. The paper by Chen *et al*. demonstrated how generalized Turing patterns (that is, Turing patterns that evolve with time) could be obtained using coupled maps.

The structure of the paper is as follows. The stability condition of synchronized state in terms of eigenvalues of the coupling matrix is discussed in Sec. 2. Then, in Sec. 3, we describe a general method to obtain coupling coefficients of a coupled map system such that it exhibits targeted synchronization within the desired subset of maps. In Sec. 4, the developed method is verified in coupled logistic maps by synchronizing a selected set of maps. By extending this technique, we store and recall images using coupled Rulkov maps in Sec. 5. A method is also developed for the storage/recall of image using multimode deviation in Sec. 6. Finally we summarize our results in Sec. 7.

## Stability of the Synchronized State in Coupled Map Lattices

In recent times, synchronization of coupled nonlinear dynamical systems^11, 12^ has become an important area of research for their applications in different fields such as neural networks^13–15^, pattern formation^10, 16, 17^, to name a few. There has been two major strands of research in this area: Theoretical investigation of different types of synchronized states and applications of synchronization to different fields. In particular, special classes of synchronizations such as partial synchronization wherein only a subset of the coupled systems exhibit synchrony^18–20^, explosive synchronization wherein there exists a positive correlation between coupling strengths of the oscillators and their natural frequencies^21, 22^ have been studied extensively. Stability of the synchronous state^11, 12, 16, 23, 24^ and its relation to the size of the coupled systems^25, 26^ have also been well studied. Another active area of research is controlling coupled oscillators to exhibit periodic oscillations where required^27^. Applications of synchronization include encoding of information in the oscillatory network of oscillators^28^ and generation of different types of (generalized) Turing patterns by a suitable choice of parameters^26, 29, 30^. For example, it is shown in these papers that one can use coupled map lattices to generate generalized Turing patterns. Classic Turing patterns are obtained by destabilizing an equilibrium point and hence the time evolution of the resultant pattern is simple. Generalized Turing patterns are obtained by destabilizing more general states including chaotic states. Hence the temporal evolution of these patterns can be chaotic. Further, the coupling is no longer restricted to diffusive coupling.

In this section, we study the stability of the synchronized state of a system of coupled maps. Consider a globally coupled map lattices (CML), where each map is coupled to every other maps, represented by

1 $$\begin{array}{c}{{\bf{x}}}^{i}(n+\mathrm{1)}=f({{\bf{x}}}^{i}(n))+\frac{1}{N}\sum _{j\mathrm{=1}}^{N}{G}_{ij}\,f({{\bf{x}}}^{j}(n)),\\ \quad \quad \quad i=1,2,\ldots ,N,\end{array}$$

where **x** ^*i*^(*n*) is the *M*-dimensional state vector of the *i*th map at the discrete time *n*, *G*_*ij*_ represents the coupling strength between *i*th and *j*th maps, and *N* is the total number of maps in the CML. If we define the homogeneous synchronized state (synchronization manifold) as **x** ^1^(*n*) = **x** ^2^(*n*) = … = **x** ^*N*^(*n*) = **x**(*n*), then the linearization of Eq. (1) around this state leads to

2 $${{\bf{z}}}^{i}(n+\mathrm{1)}={\bf{J}}({\bf{x}}(n)){{\bf{z}}}^{i}(n)+\frac{1}{N}\sum _{j\mathrm{=1}}^{N}{G}_{ij}{\bf{J}}({\bf{x}}(n)){{\bf{z}}}^{j}(n)$$

where **z** ^*i*^(*n*) denotes the deviation of the *i*th map from the synchronized state (**x**(*n*)) and **J** is the Jacobian matrix of order *M*. Following standard procedure^23^, we define an *M* × *N* matrix **S**(*n*) = (**z** ^1^(*n*) **z** ^2^(*n*) … **z** ^*N*^(*n*)). This matrix is a collection of all the deviation variables and describes the dynamics of deviation from the synchronized state. The number of columns represents the number of maps (*N*) and the number of rows represents the number of variables in each map (*M*). Then Eq. (2) can be written more compactly as

3 $${\bf{S}}(n+\mathrm{1)}={\bf{J}}({\bf{x}}(n)){\bf{S}}(n)+\frac{1}{N}{\bf{J}}({\bf{x}}(n)){\bf{S}}(n){{\bf{G}}}^{T}$$

where **G** ^*T*^ is the transpose of the coefficient matrix of order *N*. Let **e**_*i*_ be the eigenvector corresponding to the eigenvalue *λ*_*i*_ of **G** (*i* = 1, 2, …, *N*). Multiplying Eq. (3) by **e**_*i*_ and replacing **S**(*n*)**e**_*i*_ by the *M*-dimensional vector **u**_*i*_, it becomes

4 $$\begin{array}{c}{{\bf{u}}}_{i}(n+\mathrm{1)}=(1+\frac{{\lambda }_{i}}{N}){\bf{J}}({\bf{x}}(n)){{\bf{u}}}_{i}(n),\\ \quad \quad \quad i=1,2,\,\ldots ,\,N,\end{array}$$

where ${{\bf{u}}}_{i}(n)=\sum _{j\mathrm{=1}}^{N}{{\bf{z}}}^{j}(n){e}_{i}^{j}$ is a weighted sum of deviation of each map from the synchronized state (with the corresponding components of **e**_*i*_ as the weight factor). Thus Eq. (4) describes the dynamics of the weighted sum of deviations.

In order to investigate the stability of the synchronized state, we need an expression for the Lyapunov exponent (*μ*_*k*_) which can be written as^23^

5 $$\begin{array}{c}{\mu }_{k}={h}_{k}+\,\mathrm{ln}\,|1+\frac{{\lambda }_{i}}{N}|\\ \,\,k=1,2,\ldots ,M;\,i=1,2,\,\ldots ,\,N,\end{array}$$

where *h*_*k*_ represents the Lyapunov exponent of the isolated map. Intuitively, Lyapunov exponents represent the exponential rate of linear divergence from the synchronized state. The condition for the existence of homogeneous chaotic synchronized state (where all the maps are synchronized), that is, $\sum _{j\mathrm{=1}}^{N}{G}_{ij}=0$ requires that one of the eigenvalues of the coupling matrix (**G**) should equal zero. This requirement is also reflected in Eq. (4) and it is the remaining eigenvalues that determine the stability of the synchronized state. Each such non-zero eigenvalue gives rise to *M* transverse Lyapunov exponents^23^ as in Eq. (5). A negative transverse Lyapunov exponent would correspond to the case where any deviation in the corresponding direction from the synchronized state goes to zero asymptotically. For stability we require that all transverse Lyapunov exponents be negative. That is*, μ*_*max*_ = *h*_*max*_ + ln|1 + *(λ*_*i*_)/(*N*)| < 0 (for all non-zero eigenvalues) which is equivalent to the condition

6 $$\begin{array}{c}-N-N\,\exp (-{h}_{max}) < {\lambda }_{i} < -N+N\,\exp (-{h}_{max}),\\ i=1,2,\ldots ,N.\end{array}$$

## Targeted Synchronization using Single Mode Deviation

Having obtained stability conditions for the homogeneous synchronized state, we now prescribe a method for constructing the coupling matrix such that targeted synchronization is achieved. For this, we first need some preliminary results. We start by making one of the eigenvalues obtained above to fall outside the stability region of the homogeneous synchronized state and thereby allowing the system to deviate along the corresponding eigenvector (this is called single mode deviation). In particular we impose the following conditions on the eigenvalues and eigenvectors: (i) *λ*_1_ = 0 with the corresponding eigenvector **e**_1_ = (1, 1, …, 1)^*T*^, (ii) *λ*_2_ is chosen outside its stability region so that the original homogeneous synchronized state is unstable, and the corresponding eigenvector **e**_2_ is defined in such a way that the sum over all of its components is zero (This condition is to ensure that the net deviation of coupled map system from the synchronized state is zero); it is preferable to choose the value of *λ*_2_ to be close to the stability boundary to avoid numerical blow up, (iii) The remaining eigenvalues are assigned to a stable value −*N* which is the midpoint of the stable interval defined in Eq. (6) (this condition extinguishes the deviation of the system along all the eigenvectors other than **e**_2_ according to Eq. (4)), and the eigenvectors **e**_3_, **e**_4_,…, **e**_*N*_ are chosen to be a set of random orthogonal vectors, (iv) All the eigenvectors, namely, **e**_1_, **e**_2_,…, **e**_*N*_ are made orthogonal to each other.

Considering all the above conditions, we define a diagonal eigenvalue matrix **D** with diagonal elements given by (0, *λ*_2_, −*N*, …, −*N*), where *λ*_2_ is chosen outside the stability region. The general form of an orthogonal eigenvector matrix consistent with the above conditions is given by

7 $${\bf{e}}=(\begin{array}{ccccc}1 & {e}_{2}^{1} & {o}_{1}^{1} & \cdots  & {o}_{N-2}^{1}\\ 1 & {e}_{2}^{2} & {o}_{1}^{2} & \cdots  & {o}_{N-2}^{2}\\ 1 & {e}_{2}^{3} & {o}_{1}^{3} & \cdots  & {o}_{N-2}^{3}\\ \vdots  & \vdots  & \vdots  & \vdots  & \vdots \\ 1 & {e}_{2}^{N} & {o}_{1}^{N} & \cdots  & {o}_{N-2}^{N}\end{array})$$

where **o**_1_, **o**_2_, …, **o**_*N*−2_ are random orthogonal vectors. Finally the coupling matrix is obtained by the similarity transformation^10^ as

8 $${\bf{G}}={\bf{eD}}\,{{\bf{e}}}^{-1}\mathrm{.}$$

The similarity transformation results only in a change of bases and the eigenvalues remain unchanged. In particular, the eigenvalues of **G** are identical to those of **D** and hence the stability characteristics also remain the same. When the homogeneous synchronized state is destabilized in the above manner, that is, when we use the coupling matrix (8) in Eq. (1), the deviation is only along **e**_2_. From the definition of **u** it is inferred that the deviation of *i*th map in the CML is weighted with the corresponding component of **e**_2_, that is, ${e}_{2}^{i}$. *The crucial point is that the time evolution of each map is such that its deviation from the synchronized state is taking place by preserving their weight factors in* **u** *due to* Eq. (4). In other words, the time evolution of the deviation of each map should be consistent with that of the weighted sum of the deviations. Hence

9 $$\begin{array}{c}{{\bf{z}}}^{i}(n)={\bf{k}}(n){e}_{2}^{i},\quad i=\mathrm{1,}\,\mathrm{2,}\,\ldots ,\,N\quad ({\rm{or}})\\ {z}_{j}^{i}(n)={k}_{j}(n){e}_{2}^{i},\quad j=\mathrm{1,}\,\mathrm{2,}\,\ldots ,\,M,\quad i\,\mathrm{=1,}\,\mathrm{2,}\ldots ,N,\end{array}$$

where *k*_*j*_(*n*) is a proportionality constant corresponding to *j*th dynamical variable for all maps at a particular discrete time *n*.

We now use the above results for two purposes: (1) To achieve targeted synchronization wherein only a desired subset of maps are made to synchronize by means of setting the weight factors of deviations of these maps equal, that is, by assigning equal values to the corresponding components of **e**_2_. For instance, if we require to synchronize *p*, *q*, and *r*th maps, then we set ${e}_{2}^{p}={e}_{2}^{q}={e}_{2}^{r}=c$, where *c* is a common weight factor for these three maps. Here *c* is also a measure of deviation of this targeted synchronous state of these maps from the average value **x**(*n*) = (**x** ^1^(*n*) + **x** ^2^(*n*) + … + **x** ^*N*^(*n*))/*N*. *If c is taken to be zero, the targeted synchronous state remains as* **x**(*n*).

(2) More generally, to control the dynamics of each map in the coupled system in such a way that its deviation from **x**(*n*) contains the complete information about a particular pixel value of a given pattern/image. For example, if the *j*th map is assigned to carry the pixel value 156, then ${e}_{2}^{j}$ (the weight factor of the deviation of *j*th map from **x**(*n*) in defining **u**) is set to 156. Similarly all components of **e**_2_ are assigned to have the corresponding pixel value in the given pattern during the determination of *G* that encodes the entire pattern. Since *k*_*j*_(*n*) is same for all maps, if one of the components of **e**_2_ is preassigned then all other components which bear the required pattern can be retrieved from the dynamics of the CML by inverting Eq. (9).

## Targeted Synchronization in Coupled Logistic Maps

To illustrate targeted synchronization, we consider coupled logistic maps (CLM) by choosing *f*(*x*) = 1 − *ax* ^2^ in Eq. (1). The control parameter *a* is fixed at 1.9 so that it exhibits a chaotic oscillation. Logistic map (with the given parameter) was chosen only for illustration since it is a standard well-studied map. The method is applicable to maps of any dimension. For *N* = 9, the stability region of the synchronized chaos is found to be −14.205 < *λ* < −3.794. In order to construct a suitable coupling matrix *G* which ensures the targeted synchronized state, we define a 9 × 9 diagonal eigenvalue matrix *D* with diagonal elements given by: (0, −3, −9, … −9).

**Figure 1:**
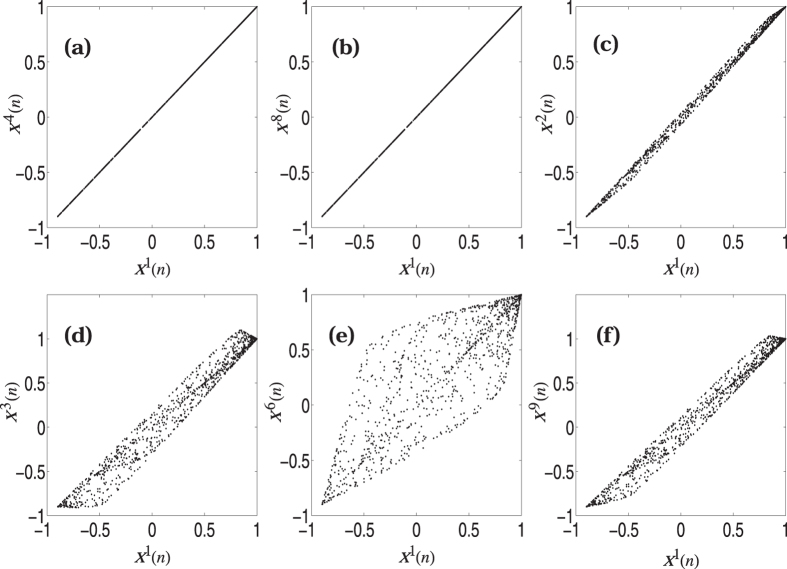
Targeted synchronization of coupled logistic maps where the synchronized value coincides with the average value *x*(*n*): (**a** and **b**) the synchronization of the first map with 4th and 8th maps, (**c**–**f**) the desynchronization of other maps (2, 3, 6 & 9) with the first map.

In this example, we target the maps 1, 4 and 8 to synchronize with the average value *x*(*n*) ( = *x* ^1^(*n*) + *x* ^2^(*n*) + … + *x* ^9^(*n*))/9) by setting the corresponding components of **e**_2_ as 0. In principle, the remaining components may take any values such that the sum of all components of **e**_2_ is zero. However, to make the study more general, we assign the value 10 identically to 2nd, 5th and 7th components of **e**_2_ to synchronize the corresponding maps to a value that is different from the average value *x*(*n*). Hence the resulting form of **e**_2_ is taken to be (0, 10, 42, 0, 10, −103, 10, 0, 31)^*T*^. Substituting this eigenvector in Eq. (7) and following the procedure discussed above, we finally obtain the coupling matrix *G* that encodes this pattern. Using this *G* in Eq. (1) (with *f*(*x*) given by the logistic map), we find that the desired targeted synchronization is achieved within the sets of {1, 4, 8} with *x*(*n*) as the synchronized state. This is shown in Fig. 1. The set of maps {2, 5, 7} have a synchronized state different from *x*(*n*) as expected.

## Image Storage and Recall using Coupled Rulkov Maps

In this section, we exploit the ability to control the dynamics of a particular map in the coupled map system to store a given pattern. We use Rulkov maps, which have been extensively used to model neurons. Rulkov maps correspond to neurons and coupling strengths correspond to synaptic strengths. Just as the human brain is thought to store memories by changing the pattern of synaptic strengths between neurons, in our model, the image is stored by changing coupling strengths between Rulkov maps. This hints at the possibility that our model could also serve as a preliminary mathematical model of how the human brain stores and recalls images.

To store a one dimensional pattern described by **e**_2_, we start with the coupled maps (1) where the dynamics of each lattice site is described by the Rulkov map

10 $$\begin{array}{c}{x}_{1}(n+\mathrm{1)}=\alpha \mathrm{/(1}+{({x}_{1}(n))}^{2})+{x}_{2}(n),\\ {x}_{2}(n+\mathrm{1)}={x}_{2}(n)-\sigma {x}_{1}(n)-\beta ,\end{array}$$

with a set of parameters *α* = 3.5, *β* = 0.0005 and *σ* = 0.001. We obtain similar behaviour with other sets of parameters, except that the stability region is different in these cases.

**Figure 2:**
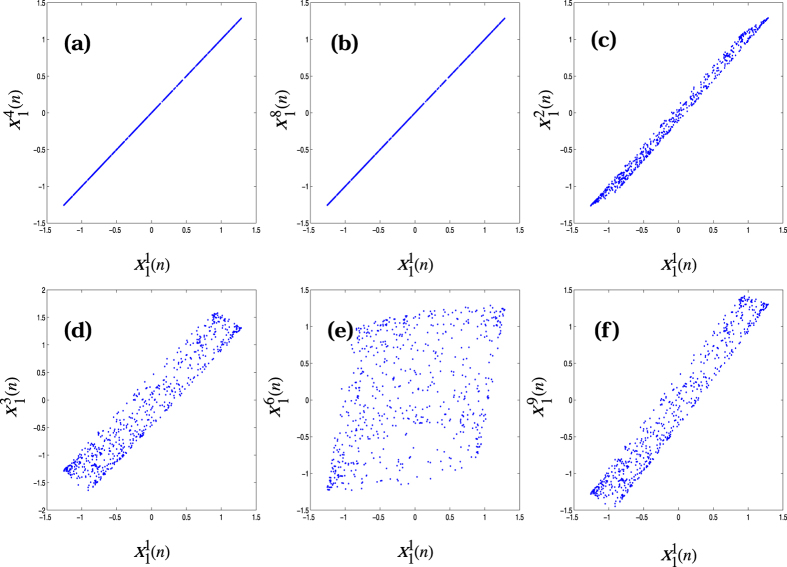
Targeted synchronization of coupled Rulkov maps where the synchronized value coincides with the average value *x*_1_(*n*): (**a** and **b**) the synchronization of the first map with 4th and 8th maps, (**c**–**f**) the desynchronization of other maps (2, 3, 6 & 9) with the first map.

Before proceeding with image storage and recall, we first demonstrate that Rulkov maps also exhibit targeted synchronization. We use a system of 9 coupled Rulkov maps. As in the case of coupled logistic maps, we again target the maps 1, 4 and 8 to synchronize with the average value *x*_1_(*n*) $(={x}_{1}^{1}(n)+{x}_{1}^{2}(n)+\ldots +{x}_{1}^{9}(n\mathrm{))/9}$). This is shown in Fig. 2.

We now illustrate the storage and recall of a simple 1-dimensional pattern represented by the set {142, 10, 200, 58, 96, 3, 171}. For this purpose, we use a system of coupled Rulkov maps of size *N* = 9 with the coupling matrix determined by following the procedure described in Sec. 3. During the determination of the coupling matrix, it should be taken into account that the stability region for the synchronized state is −17.01 ≤ *λ* ≤ −0.99 and we should assign **e**_2_ = (142, 10, 200, 58, 96, 3, 171, −681, 1)^*T*^. It should also be noted here that two additional components have been appended in **e**_2_, the first one is to make the sum of components of **e**_2_ zero and the second is to obtain the proportionality constant involved in Eq. (9), namely, *k*_1_(*n*) or *k*_2_(*n*). The diagonal elements of *D* is set to (0, 1, −9, −9, −9, −9, −9, −9, −9). Following the procedure outlined earlier, the given pattern is stored in the coupled Rulkov maps through a set of coupling coefficients, that is, as a coupling matrix *G*.

**Figure 3:**
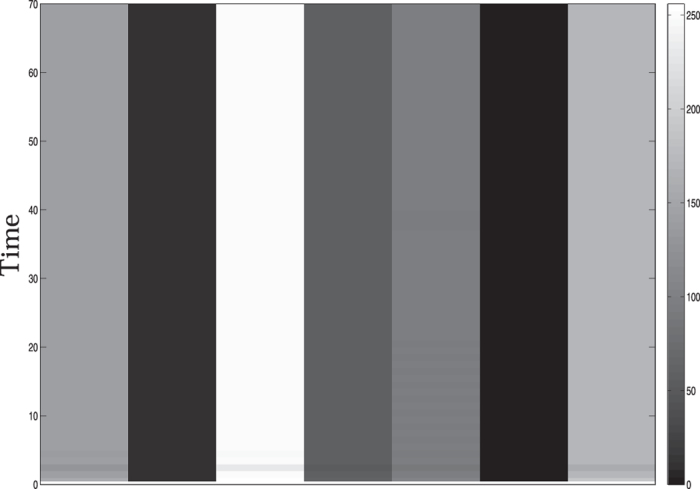
Splined (in *x* direction) image of one dimensional pattern {142, 10, 200, 58, 96, 3, 171} decoded from the globally coupled Rulkov maps with *α* = 3.5, *β* = 0.0005, *σ* = 0.001 and *N* = 9.

The deviation of *i*th map from the synchronized state *x*_1_(*n*), that is, ${z}_{1}^{i}(n)={x}_{1}^{i}(n)-{x}_{1}(n)$ is evaluated using the map defined by the dynamical Eq. (10) for the purpose of usage in Eq. (9). Finally, the pattern recalled from the dynamics of coupled Rulkov maps using Eq. (9) and its splined (in *x* direction) image is shown Fig. 3. Here *y* axis represents time evolution of the pattern which is found to be invariant after transient. It has been verified that equal values in the components of e_2_ result in synchronization of the corresponding maps in the coupled dynamical systems, that is, a set of maps bearing same pixel value exhibit targeted synchronization.

## Image Storage and Recall using Multimode Deviation

So far, we considered only the single mode deviation of a coupled map system in the study of targeted synchronization and image storage/recall. We now describe an effective method to encode and recall a *p* × *p* pixel image using multimode deviations (where we destabilize the synchronized state along more than one eigenvector). This permits us to use a single globally coupled system, whose coupling coefficients have a 2-d topology, to encode a 2-d image. It should be emphasized that this is a single globally coupled system of maps with a 2-d coupling topology and not a sequence of 1-d maps. The method uses a coupled map system of size *N* = 2*p* + 1 (with a 2-d topology defined by *N* × *N* coupling coefficients) to store *p* × *p* pixels. Encoding *p* × *p* pixels would normally require the system to be of size *p* implying that the coupling coefficient matrix should be of size *p* × *p*. However, for the purpose of decoding, as we shall see, the coupled system is taken to be of size 2*p* + 1, the minimum size required to both store and recall *p* × *p* pixels. In principle, this system can store *p* ^2^ independent images.

In this method, we allow the coupled map system to deviate from its synchronized state along *p* of 2*p* + 1 eigenvectors by choosing the corresponding eigenvalues to fall outside the stability region of synchronized state so that the diagonal elements of *D* become (0, *λ*_2_, *λ*_3_, *λ*_*p*+1_, −(2*p* + 1), −(2*p* + 1), −(2*p* + 1)), where *λ*_2_, *λ*_3_, …, *λ*_*p*_ and *λ*_*p*+1_ are all unstable eigenvalues. It is straightforward to obtain the coupling matrix *G* from (8) by choosing the (2*p* + 1) eigenvectors as follows. The first eigenvector is taken to be **e**_1_ = (1, 1, …, 1). The components of next *p* eigenvectors **e**_2_, **e**_3_, **e**_*p*+1_, corresponding to the *p* unstable eigenvalues, are specified as follows. The first *p* components of the *i*th eigenvector (*i* = 2, …, *p* + 1) are taken to be the *p* pixels corresponding to the (*i* − 1)th row of the image. The next *p* components of each of these eignevectors are chosen randomly but with the condition that the eigenvectors are orthogonal to one another. Finally, a (2*p* + 1)th component is appended to each eigenvector such that the sum over all components of that eigenvector becomes zero. The last *p* eigenvectors (corresponding to the last *p* stable eigenvalues) are chosen to be random orthogonal eigenvectors. The coupling matrix *G* (of order *N* × *N*), obtained in this manner, directly stores a two dimensional pattern of pixel size *p* × *p*.

The image can be recalled from the evolution of the single globally coupled system as follows. The assumption that we made, namely, that the system can deviate along *p* eigenvectors from the homogeneous synchronized state introduces *p* extra constraints in the transverse evolution of each map. Because of these constraints, the deviation of the *i*th map is restricted to the directions of ${e}_{2}^{i},{e}_{3}^{i},\,\ldots ,\,{e}_{p}^{i}$ and ${e}_{p+1}^{i}$ such that

11 $${z}_{m}^{i}(n)=\sum _{j\mathrm{=2}}^{p+1}{k}_{m}^{j}(n){e}_{j}^{i};\quad m=\mathrm{1,}\,\mathrm{2,}\,\ldots ,\,M\quad {\rm{and}}\quad i=\mathrm{1,}\,\mathrm{2,}\ldots ,N\mathrm{.}$$

By solving an appropriate number of equations defined by (11), we can recall the pattern $({e}_{j}^{i};\,j=\mathrm{2,}\,\mathrm{3,}\ldots ,p+\mathrm{1,}\,i=\mathrm{1,}\,\mathrm{2,}\ldots ,p)$ from the dynamics of the coupled map as described below. It is important to note that ${k}_{m}^{j}(n)$ is independent of *i*, that is, its value is the same for all maps. Thus, for a fixed *m*, the substitution of *i* = *p* + 1, *p* + 2, …, 2*p* in Eq. (11) gives rise to a set of *p* simultaneous algebraic equations for ${k}_{m}^{2}(n),\,{k}_{m}^{3}(n),\ldots ,{k}_{m}^{p}(n)$ and ${k}_{m}^{p+1}(n)$. This set of simultaneous equations has a unique solution, since each coefficient involved in these equations (${e}_{j}^{i};j=\mathrm{2,}\ldots ,p+1$ and *i* = *p* + 1, *p* + 2, …, 2*p*) has a preassigned random value. The same procedure can be followed to obtain *p* − 1 other such sets of *k* evaluated at different discrete times after the transient. This gives us ${k}_{m}^{2}(n+\mathrm{1),}{k}_{m}^{3}(n+\mathrm{1),}\ldots ,{k}_{m}^{p+1}(n+\mathrm{1)}$; $\ldots$ ${k}_{m}^{2}(n+p-\mathrm{1),}{k}_{m}^{3}(n+p-\mathrm{1),}\ldots ,{k}_{m}^{p+1}(n+p-\mathrm{1)}$.

Our primary goal is to determine ${e}_{j}^{i}$’s (*j* = 2, *p* + 1 and *i* = 1, 2, …, *p*) which actually represent the image that was encoded in *G*. To obtain, for example ${e}_{j}^{1}$, *j* = 2, …, *p* + 1, we solve a set of *p* simultaneous equations obtained by setting *i* = 1 in Eq. (11) for *p* discrete times after discarding the transient. Similarly, ${e}_{j}^{2}$; *j* = 2, …, *p* + 1 is determined by solving another set of *p* simultaneous equations obtained by substituting *i* = 2, for *p* iterations in Eq. (11). Following this procedure, we get all the remaining components of the eigenvectors ***e***_*j*_, *j* = 2, 3, …, *p* + 1 thus permitting us to recall the image.

**Figure 4:**
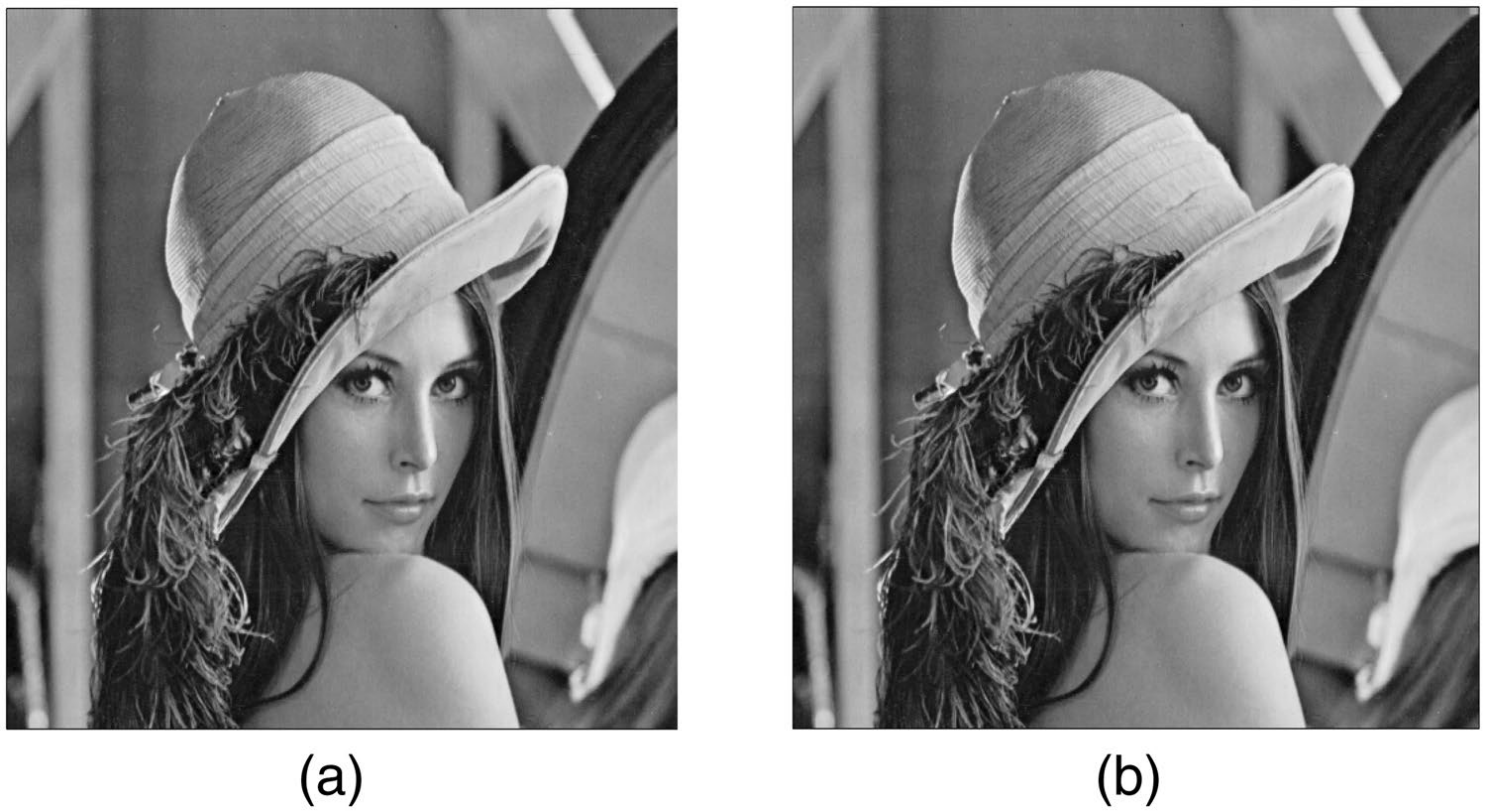
Images of Lena: (**a**) Original image and (**b**) Image decoded from a globally coupled system of Rulkov maps with *α* = 3.5, *β* = 0.0005, *σ* = 0.001 and *N* = 1025.

To illustrate this method, we consider the image of Lena with 512 × 512 pixels resolution. The size of the coupled Rulkov map system is set to *N* = 2*p* + 1 = 1025 which gives the stability range of the synchronized state to be −1937.1 < *λ* < −112.1 for the parameter set *α* = 3.5, *β* = 0.0005, *σ* = 0.001. Following the procedure discussed above, we construct the coupling matrix *G*. The image is recalled from a set of 1025 coupled Rulkov maps which are coupled through *G* using Eq. (11). The final recalled image is found to be exactly the same as the original image as shown in Fig. 4.

## Discussions

In this paper, using targeted synchronization and coupled Rulkov maps, we have explicitly demonstrated a mathematical model for storage and recall of realistic 2-d images. We used a single globally-coupled system with multimode deviation for this purpose. Our model has its limitations. There is no guarantee that the final pattern can be predicted from the most unstable eigenvectors (modes) of the model since this is only a linear model. This would actually depend on the basin of attraction of the stored images in the full nonlinear map.

Next, we briefly discuss the relationship of our model to storage and recall of images in the brain. There has been much work on the mechanisms by which the brain encodes and decodes memories (images). The general contours of such mechanisms are well understood and involve long-term potentiation, synchronous firing of neurons, and the pattern of synaptic weights^1–4^. Storage of images depends on the plasticity of synaptic connections. In particular, synchronous firing of a group of neurons can lead to an increase in the synaptic strengths between members of this group through long term potentiation. Storage of images occurs through this pattern of synaptic weights. This allows the brain to internally recreate the pattern of activity later and thereby recall the image. Further, it has been hypothesized^31^ that synchronization and synaptic plasticity may form a positive feedback loop where extended periods of synchronization mediated communication enhances synaptic strengths and the increased synaptic strengths in turn facilitate synchronization within the group.

Our model mimics two important aspects of image storage and recall in the brain as described above: synchronization and synaptic modification of weights (which corresponds to changes in coupling strengths in our model). In this sense, our model could be thought of as a bio-inspired or a neuromorphic image encoding mechanism. But one could turn this around and hypothesize that the brain could conceivably use a similar coupled system of neurons as proposed by us to store and recall memories. Needless to say, our model can not be directly applicable. For example, Rulkov maps are an approximation for the real neurons. This approximation focuses on capturing only the dynamical behaviour of the neuron in a simple fashion at the cost of reproducing the detailed biophysical mechanisms^9^. In its defense, the all important dynamical behaviour of action potentials is well-captured by the approximation. It remains to be seen how a more realistic model of the neuron will impact our mechanism for storage and recall. Further, we have considered only a simplistic coupling between neurons. A more realistic synaptic coupling could also alter our conclusions and needs to be considered. Therefore much additional work would be required to clarify the applicability of our model to the human brain.

### Data availability

All data generated or analysed during this study are included in this published article.

## References

[1] Dayan, P. & Abbott, L. F. *Theoretical Neuroscience* (The MIT Press, Massachusetts, 2001).

[2] Miltner WHR, Braun C, Arnold M, Witte H, Taub E (1999). Coherence of gamma-band eeg activity as a basis for associative learning. Nature.

[3] Axmacher N, Mormann F, Fernandez G, Elger CE, Fell J (2006). Memory formation by neuronal synchronization. Brain Res. Rev..

[4] Jutras MJ (2010). & Buffalo, E. A. Synchronous neural activity and memory formation. Curr. Opin. Neurobiol..

[5] Hopfield JJ (1982). Neural networks and physical systems with emergent collective computational abilities. Proc. Natl. Acad. Sci. USA.

[6] Hopfield JJ (1984). Neurons with graded response have collective computational properties like those of two-state neurons. Proc. Natl. Acad. Sci. USA.

[7] Wang Z, Ma Y, Cheng F, Yang L (2010). Review of pulse-coupled neural networks. Image Vis. Comput..

[8] Jones JP, Palmer LA (1987). An evaluation of the two-dimensional gabor filter model of simple receptive fields in cat striate cortex. J. Neurophysiol..

[9] Girardi-Schappo M, Tragten-berg MHR, Kinouchi O (2013). A brief history of excitable map-based neurons and neural networks. J. Neurosci. Meth..

[10] Chen Y, Rangarajan G, Ding M (2006). Stability of synchronized dynamics and pattern formation in coupled systems: Review of some recent results. Commun. Nonlinear Sci. Numer. Simul..

[11] Pecora LM, Carroll TL (1990). Synchronization in chaotic systems. Phys. Rev. Lett.

[12] Pecora LM, Carroll TL (1991). Driving systems with chaotic signals. Phys. Rev. A.

[13] Hansel D, Sompolinsky H (1993). Solvable model of spatiotemporal chaos. Phys. Rev. Lett..

[14] Hansel D (1996). Synchronized chaos in local cortical circuits. Int. J. Neur. Syst..

[15] Pasemann F (1999). Synchronized chaos and other coherent states for two coupled neurons. Physica D.

[16] Rangarajan G, Chen Y, Ding M (2003). Generalized turing patterns and their selective realization in spatiotemporal systems. Phys. Lett. A.

[17] Kliakhandler IL (2000). Selection of scales in pattern-forming dynamics. Phys. Rev. E.

[18] Lim W, Kim S-Y (2005). Mechanism for the partial synchronization in three coupled chaotic systems. Phys. Rev. E.

[19] Yu D, Parlitz U (2008). Partial synchronization of chaotic systems with uncertainty. Phys. Rev. E.

[20] Poel W, Zakharova A, Schöll E (2015). Partial synchronization and partial amplitude death in mesoscale network motifs. Phys. Rev. E.

[21] Zhang X, Hu X, Kurths J, Liu Z (2013). Explosive synchronization in a general complex network. Phys. Rev. E.

[22] Pinto RS, Saa A (2015). Explosive synchronization with partial degree-frequency correlation. Phys. Rev. E.

[23] Rangarajan G, Ding M (2002). Stability of synchronized chaos in coupled dynamical systems. Phys. Lett. A.

[24] Amritkar RE, Rangarajan G (2009). Stability of multicluster synchronization. Int. J. Bifurcat. Chaos.

[25] Palaniyandi P, Rangarajan G (2007). Critical lattice size limit for synchronized chaotic state in one- and two-dimensional diffusively coupled map lattices. Phys. Rev. E.

[26] Palaniyandi P, Muruganandam P, Lakshmanan M (2005). Desynchronized wave patterns in synchronized chaotic regions of coupled map lattices. Phys. Rev. E.

[27] Prasad A, Dhamala M, Adhikari BM, Ramaswamy R (2010). Targeted control of amplitude dynamics in coupled nonlinear oscillators. Phys. Rev. E.

[28] Wang S, Zhou C (2009). Information encoding in an oscillatory network. Phys. Rev. E.

[29] Chen P, Viñals J (1997). Pattern selection in faraday waves. Phys. Rev. Lett..

[30] Kenig E, Lifshitz R, Cross MC (2009). Pattern selection in parametrically driven arrays of nonlinear resonators. Phys. Rev. E.

[31] Fell J, Axmacher N (2011). The role of phase synchronization in memory processes. Nature Rev. Neurosci..

